# When More Means Less: The Prognosis of Recurrent Acute Myocardial Infarctions

**DOI:** 10.3390/jcm10245889

**Published:** 2021-12-15

**Authors:** Ygal Plakht, Harel Gilutz, Arthur Shiyovich

**Affiliations:** 1 Department of Nursing, Faculty of Health Sciences, Ben-Gurion University of the Negev, Beer-Sheva 8410501, Israel; 2 Department of Emergency Medicine, Soroka University Medical Center, Beer-Sheva 8489501, Israel; 3 Faculty of Health Sciences, Goldman Medical School, Ben-Gurion University of the Negev, Beer-Sheva 8410501, Israel; Gilutz@bgu.ac.il; 4 Department of Cardiology, Rabin Medical Center, Petah Tikva 4941492, Israel; arthur.shiyovich@gmail.com; 5 Sackler Faculty of Medicine, Tel Aviv University, Tel Aviv 6139001, Israel

**Keywords:** recurrent acute myocardial infarction, mortality, prognosis, follow-up study

## Abstract

Recurrent acute myocardial infarctions (AMI) are common and associated with dismal outcomes. We evaluated the clinical characteristics and the prognosis of AMI survivors according to the number of recurrent AMIs (ReAMI) and the time interval of events (TI). A retrospective analysis of patients who survived following hospitalization with an AMI throughout 2002–2017 was conducted. The number of ReAMIs for each patient during the study period was recorded and classified based on following: 0 (no ReAMIs), 1, 2, ≥3. Primary outcome: all-cause mortality up to 10 years post-discharge from the last AMI. A total of 12,297 patients (15,697 AMI admissions) were analyzed (age: 66.1 ± 14.1 years, 68% males). The mean number of AMIs per patient was 1.28 ± 0.7; the rates of 0, 1, 2, ≥3 ReAMIs were 81%, 13.4%, 3.6% and 1.9%, respectively. The risk of mortality increased in patients with greater number of AMIs, HR = 1.666 (95% CI: 1.603–1.720, *p* < 0.001) for each additional event (study group), attenuated following adjustment for potential confounders, AdjHR = 1.135 (95% CI: 1.091–1.181, *p* < 0.001). Increased risk of mortality was found with short TI (<6-months), AdjHR = 2.205 (95% CI: 1.418–3.429, *p* < 0.001). The risk of mortality following AMI increased as the number of ReAMIs increased, and the TI between the events shortened. These findings should guide improved surveillance and management of this high-risk group of patients (i.e., ReAMI).

## 1. Introduction

Throughout the recent decades, multiple significant advancements in the management of patients with an acute myocardial infarction (AMI) were introduced and led to improved short- and long-term outcomes [1,2,3,4]. However, AMI survivors remain at increased risk of various adverse outcomes, including a recurrent AMI (ReAMI) [5,6]. Approximately 10% of patients are at risk of developing a ReAMI, accounting for about 200,000 cases per year in the United States [5,6,7,8,9,10]. Furthermore, ReAMIs are reported to be associated with worse outcomes [5,6,9,10]. However, previous reports often consisted of relatively short follow-up for ReAMIs or outcomes, were not contemporary and did not evaluate patients with multiple ReAMIs. The objective of the current study was to evaluate the clinical characteristics and the prognosis of AMI survivors with ReAMIs according to the number of events and the time interval between events (TI).

## 2. Materials and Methods

### 2.1. Study Population

The present study includes a retrospective analysis of patients who survived following hospitalization with an AMI from Soroka University Medical Center (SUMC) throughout 2002–2017. SUMC is a tertiary referral center (~1200 beds), singly serving the metropolitan area of Beer-Sheva, southern Israel (more than 500,000 residents). The last hospital admission during the period of data collection was defined as the index event. Patients were excluded due to the following criteria: (1) patients who were not residents of Southern District of Israel, (2) patients with a history of an MI prior to the earliest AMI event throughout the study period, (3) patients who died during their first admission during the study period and (4) AMI events that occurred within 28 days or less after a previous AMI. Such consecutive events might not represent a new separate event, but rather episodes of care related to the first event [11,12]. The ethics committee of SUMC approved the study, which was performed in accordance with the Helsinki Declaration.

### 2.2. Data Sources and Classifications

Data were obtained from the electronic medical files of SUMC (baseline characteristics) and the Ministry of the Interior Population Registry (mortality data). Baseline data of the index event included patients’ demographic and clinical characteristics, laboratory, echocardiographic and angiographic data and management, as previously reported for the Soroka Acute Myocardial Infarction (SAMI) project [4]. Most baseline variables relating to past medical history were acquired using the International Classification of Diseases, Ninth Revision, Clinical Modification (ICD-9-CM) discharge codes. Specifically, AMI diagnosis was identified based on the ICD-9-CM codes: ST elevation MI (STEMI): 410.0 *–410.6 * and non-ST elevation MI (NSTEMI): 410.7 *–410.9 *. In addition to the diagnostic codes, the diagnoses of anemia were grouped together with low hemoglobin blood levels. Patients were defined as having significant renal failure if they were either on hemodialysis/peritoneal dialysis or had an estimated glomerular filtration rate (eGFR) of <30 mL/min/1.73 m^2^. The diagnosis of diabetes mellitus comprised of hemoglobin A1C levels ≥6.5%, and dyslipidemia was considered if low-density lipoprotein level was ≥100 mg/dL. Severe left ventricular (LV) dysfunction was defined as a left ventricular ejection fraction (LVEF) of <30%.

### 2.3. Study Groups

Patients were classified based on the number of AMI events throughout the study period as following: 0 (no ReAMI), 1, 2 and ≥3. Additional analyses of the outcome ReAMIs, according to various TI of events, categorized as <0.5 year, 1, 2, 3, 4 and ≥5 years, were performed.

### 2.4. Follow-Up and Outcomes

The primary outcome was all-cause mortality. The follow-up period to the primary outcome extended up to 10 years following the index event, with the last update being on 31 May 2021.

### 2.5. Statistical Analysis

Statistical analysis was executed using IBM SPSS Statistics 26 software. Patient characteristics were displayed as mean and standard deviation (SD) for continuous variables and n and percent for the categorical data. Baseline characteristics were compared between the study groups using Chi-square test for linear trend and analysis of variance (ANOVA) for linear trend. Outcomes were compared between the study groups using the survival approach. The univariate analysis compared the risk of mortality with creation of survival functions (Kaplan–Meier) and using the log-rank test. In addition, regression analysis (Cox regression) was performed in order to estimate the relative risk for long-term mortality for the study groups. In this analysis, the group of patients with no ReAMI served as the reference group. The multivariate analysis included Cox regression models. The parameters were introduced to the multivariate models using the stepwise method. The results of the models were presented as hazard ratios (HR)/adjusted hazard ratios (AdjHR) with 95% confidence intervals (CI). For each test, *p* < 0.05 was considered as statistically significant.

## 3. Results

### 3.1. Study Population and Groups

Throughout the study period, a total of 17,610 patients with at least one AMI were identified. Patients were excluded due to the following reasons: 2606 were not residents of the Southern District of Israel, 1627 had a prior AMI before the first AMI recorded during the study period, and 1080 died during the first admission. Thus, the final cohort for this study included 12,297 patients. The study flowchart is presented in the Supplementary Materials, Figure S1.

During the period of data collection, there were a total 15,697 admissions with an AMI. The mean number of AMIs per patient was 1.28 ± 0.7. The mean number of AMIs per patient among those who had a ReAMI was 1.46 ± 0.91. Overall, 9973 (81%) patients did not have a ReAMI, 1647 (13.4%) had one ReAMI, 446 (3.6%) had two and 231 (1.9%) had three or more ReAMIs. The distribution of the number of AMIs is presented in Table S1 of the Supplementary Materials.

The baseline characteristics of the study cohort (documented at the index hospitalization) according to the study groups are presented in Table 1. Patients in the groups with greater number of AMI events were older, with a higher proportion of women and minorities, and were more likely to present as NSTEMI compared with patients in the groups with lower number of AMI events. Furthermore, they had higher prevalence of most cardiovascular risk factors, excluding obesity and family history of ischemic heart disease, which were more prevalent among patients with lower number of AMI events. Moreover, patients with more ReAMI events had a more severe coronary artery disease, greater rate of congestive heart failure and severe left ventricular dysfunction. Additionally, they had higher prevalence of some non-cardiovascular comorbidities (i.e., chronic obstructive pulmonary disease (COPD), neurological disorders and anemia).

### 3.2. Follow-Up and Outcomes

**Figure 1:**
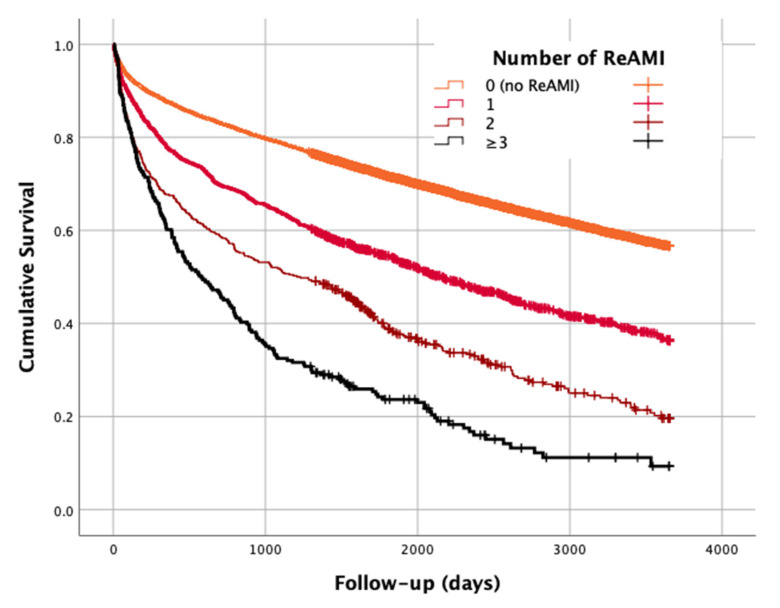
Survival function—cumulative mortality by the category of number of recurrent acute myocardial infarction (ReAMI) events. Log rank test, *p* < 0.001.

The median post-discharge follow-up was 2200 days (~6 years). During this period, 5278 (42.9%) patients died (cumulative mortality 0.480). Mortality rates (cumulative mortality) were significantly higher as the number of admissions with AMI increased: 38.6% (cumulative mortality of 0.433), 56.3% (0.636), 70.4% (0.804) and 82.7% (0.907) for the groups of 0, 1, 2 and ≥3 ReAMIs, respectively (*p*-for-trend < 0.001). Figure 1 presents the Kaplan–Meier survival functions for each study group.

**Figure 2:**
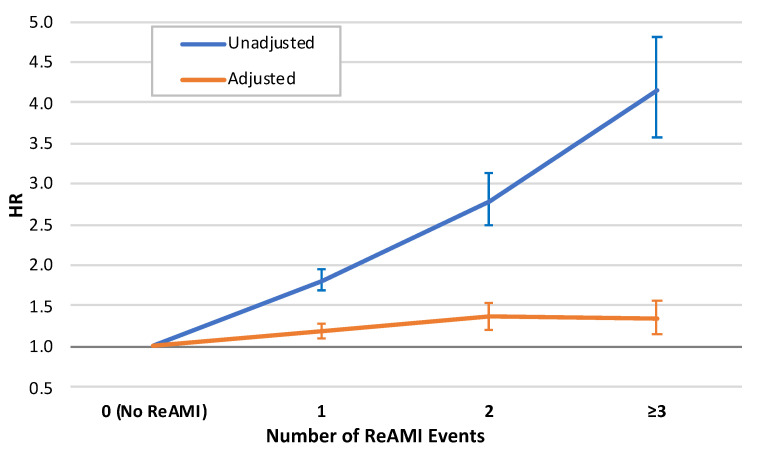
Relative risk for long-term mortality by the number of recurrent acute myocardial infarction (ReAMI) events. Blue line—unadjusted; red line—adjusted for the baseline characteristics (see Table 2 for the multivariate model). ReAMI—recurrent acute myocardial infarction, HR—hazard ratio.

The relative risks (HRs) for mortality for the groups of 1, 2 and ≥3 ReAMI events were: 1.815 (95% CI: 1.689–1.950), 2.783 (95% CI: 2.480–3.123) and 4.143 (95% CI: 3.579–4.794), respectively, as compared with the reference group (*p* < 0.001 for each) (Figure 2, blue line). With an increase in one group in the number of AMIs, the unadjusted HR for mortality was 1.666 (95% CI: 1.603–1.720, *p*-for-trend < 0.001).

### 3.3. Multivariable Analysis

Following multivariate adjustment to potential confounders (Table 2 and Figure 2, red line), AdjHRs for mortality for the groups were: 1.181, 1.362 and 1.336 for 1, 2 and ≥3 ReAMI events, respectively, as compared with the reference group. The AdjHR for mortality with an increase in one group in the number of AMIs was 1.135 (95% CI: 1.091–1.181, *p*-for-trend < 0.001).

### 3.4. Sub-Group Analysis

In order to evaluate the risk of mortality according to the various TIs, the groups of 2 ReAMIs and ≥3 ReAMIs were merged into the category of ≥2 ReAMIs (due to the small size of the latter group). The distribution of the TIs by the number of the AMI groups are presented in Supplementary Table S2.

The rates of the TIs between the AMIs in the different AMI number groups are presented in Supplementary Table S2. For every category of the number of AMIs, a significant trend (*p*-for-trend < 0.001 for each) of decrease in the risk of mortality was found when the TI increased. However, when two events occurred within half a year or less, the risk of mortality was greater compared with 0.5–1-year TI, borderline statistical significance (*p* = 0.053).

**Figure 3:**
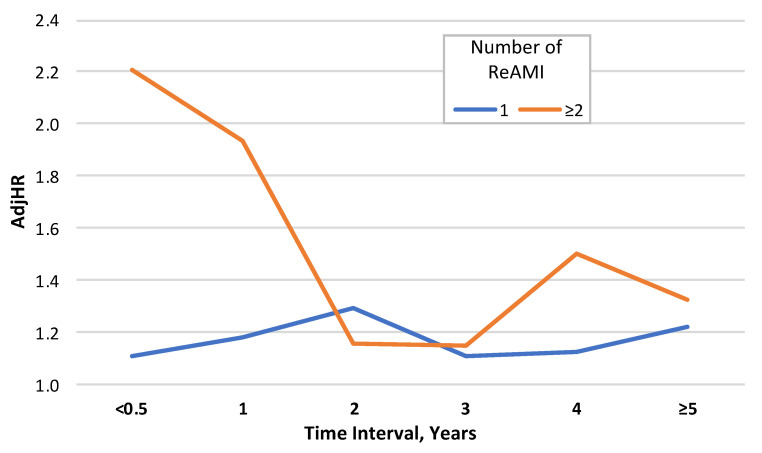
The adjusted relative risks for mortality according to the number of recurrent acute myocardial infarction (ReAMI) events * and the time intervals (see Table S3 for the full multivariable model). * Reference group—no recurrent acute myocardial infarction (ReAMI) events. AdjHR—adjusted hazard ratio.

The adjusted risks for mortality according to the number of AMI events and TIs are presented in Figure 3 and Supplementary Table S3. Following adjustment, the highest risk of long-term mortality was observed for ≥2 ReAMIs that occurred within less than 0.5 year: AjdHR = 2.205 (95% CI: 1.418–3.429) and 0.5–1-year: AjdHR = 1.933 (95% CI: 1.448–2.580), (*p* < 0.001 for each) and was significantly lower for longer TIs (as compared with the group with no ReAMI). However, no significant trend for relative risk of mortality related to TI in the group with 1 ReAMI event was found.

## 4. Discussion

The present study evaluated patient characteristics and outcomes in patients with ReAMIs according to the number of events and their TI. The main findings include: (1) the risk of mortality increased significantly with the increase in the number of AMIs, especially ≥3 ReAMI events; (2) following adjustment to potential confounders, the risk of mortality with ReAMI attenuated, yet it remained significantly increased; and (3) considering the TI of events, the highest risk was when ≥2 ReAMIs occurred during a period of less than 1 year.

The rate of patients with ReAMI in the current study (≥2 admissions with an AMI) was 18.9%: higher compared with some previous studies [5,6,11,13,14], nearly identical to a study by Viveiros et al. [15] and lower from that reported by Motivala et al. [9]. These differences stem mostly from the length of the follow-up period and the methodology used in the current study, which focused on analyzing patients according to the exact number of AMIs and not only on separating first events from ReAMIs. Additional reasons could be the real-life, highly unrepresentative cohort, with period of inclusion beginning almost two decades ago (higher rate of MACE, including ReAMIs, at that period) [4,16].

The finding that patients with ReAMI are older, with higher prevalence of most cardiovascular risk factors and more severe coronary disease, yet a decreased rate of revascularization, is also consistent with previous reports [5,6,11,13,14]. However, the current study also adds analysis with the number of ReAMIs that display a gradual (“dose response”-like) response to most variables.

The finding of this study that the risk of mortality increased significantly with the increase in the number of ReAMIs is in line with the findings of previous studies [5,6,11,13,14,17]. Although Motivala et al. [9] reported similar in-hospital mortality among patients with ReAMI vs. those with a first AMI, mortality six months following discharge was significantly higher among patients with a ReAMI. This study extends previous literature in the investigation of more than one ReAMI and demonstrates a gradual increase in risk with every event (considering also the TI between the events), the prolonged follow-up period, the unrepresentative population and the relatively high number of investigated confounders used for adjustment and indeed attenuating the independent risk significantly. The reasons for the increased risk of mortality with increased numbers of AMIs are largely unknown. However, several potential explanations can be suggested. First, patients with ReAMIs are older, with worse risk profile and higher prevalence of cardiovascular risk factors, and both cardiac and non-cardiac comorbidities, that increase their risk. Although we adjusted for age and most risk factors, some unaccounted-for comorbidities probably still contribute to their increased risk. Second, these patients were less intensively treated with a more conservative approach, which could also accelerate earlier recurrence [18]. The latter mechanism is also supported by previous literature showing worse outcome for patients presenting with apparently a first AMI but diagnosed with a previous silent AMI that was not treated [19]. Furthermore, with the increasing number of ReAMI, we saw a trend of decreasing number of coronary angiographies and percutaneous coronary interventions (PCI). Although the latter might be partially explained by a wider use of coronary artery bypass graft (CABG) with increasing ReAMIs, we believe this somewhat conservative management also stems from higher rate of NSTEMI, older and more frail patients, greater rate of chronic kidney disease and the fact that the study includes patients over a long period of time, starting from almost two decades ago, when a conservative approach was more prevalent. Third, ReAMI is often associated with reduced adherence with guidelines and compliance with optimal medical therapy, despite the strong evidence supporting the efficacy of such therapy, specifically in patients with ReAMI [5,20,21,22]. Moreover, although studies exploring the specific therapy of patients with ReAMI are insufficient, most recommendations support a stricter approach in follow-up and control of cardiovascular risk factors [23,24]. Fourth, the more severe coronary disease and particularly the increased prevalence of multivessel disease (as found in ReAMIs in the current study) have been reported as risk factors for ReAMI [6,16].

The increased risk with every ReAMI (which were usually chronologically later in our cohorts than initial events) should also be considered in the context of significantly improved outcomes in patients with AMI throughout the last decades, following major upgrades in therapy [1,2,3,4]. Thus, this risk might actually be underestimated in the current study.

The finding of the current study that the risk of mortality increases with more AMIs within shorter TI is in line with previous studies showing worse short-term mortality in cases of ReAMI [5,15,18,25]. This could be explained by the higher rate of stent thrombosis, which is associated with dismal outcomes, particularly during the relatively early period [6,15]. Nevertheless, non-stent-related ReAMI is also more common during the early period [15]. Additional reasons explaining worse outcomes when ReAMI occur during the early period following AMI include increased rates of various potentially lethal complications (e.g., mechanical complications), increased vulnerability shortly following hospitalization, delayed prescription filling and increased excessive inflammation, either as a response to the AMI or for other reasons [26,27,28,29,30].

### Limitations

Our findings need to be interpreted in the context of certain limitations. First, our study is retrospective and observational and shares the limitations of such design. Second, although our multivariate models included adjustment for multiple potential confounders, unaccounted-for confounders might still affect the observed differences between the groups. Third, we did not have information regarding the quality of care upon discharge and thereafter, in particular the rate of prescription and usage of guideline-recommended medications (e.g., stating, anti-platelet agents, angiotensin-converting enzyme (ACE) inhibitors, etc.). Fourth, the causes of death (specifically cardiac vs. non-cardiac) were not included in the current study and might have resulted in underestimation of the rates of ReAMI. Furthermore, it is possible that some of the mortalities, especially sudden death, were actually a ReAMI which was not counted as such in the current study. Fifth, silent AMIs were not systematically evaluated and recorded in the current study, which might have underestimated the rate of ReAMI. Sixth, as we used the ICD-9-CM diagnosis codes for most of the baseline characteristics, consequently, we relied on the treating physicians’ partial bias (i.e., diagnosis or coding bias), which might have occurred in some cases. However, we felt that overruling and “second guessing” the treating physician in such cases might introduce more inaccuracies. Seventh, we excluded ReAMIs within 28 days or less from the previous events, as they might not have represented a separate event; however, this might have underestimated the incidence of ReAMI in our cohort.

## 5. Conclusions

The risk of mortality following AMI increases with the increase in the number of ReAMI events and shorter TI of AMIs, with the highest risk being when ≥3 AMIs occur during a period of less than 1 year. These findings should guide more intense surveillance and management of this high-risk group of patients (i.e., ReAMI with short term), both upon hospital admission and post-discharge. Further investigation of unique strategies and treatment goals for patients with ReAMI are warranted to improve their outcomes.

## Figures and Tables

**Table 1:** The baseline characteristics of the study population (regarding to the index admission), by the number of recurrent acute myocardial infarction (ReAMI) events.

Characteristic	Number of ReAMIs				Total	*p*-for-Trend
	0	1	2	≥3		
*n*	9973	1647	446	231	12,297	
Demographics						
Age, Mean (SD)	65.19 (14.10)	69.18 (13.82)	70.96 (13.32)	73.01 (11.99)	66.08 (14.13)	<0.001
<65	5091 (51.0)	660 (40.1)	148 (33.2)	64 (27.7)	5963 (48.5)	<0.001
65–75	2181 (21.9)	377 (22.9)	118 (26.5)	53 (22.9)	2729 (22.2)	
≥75	2701 (27.1)	610 (37.0)	180 (40.4)	114 (49.4)	3605 (29.3)	
Sex, Males	6833 (68.5)	1101 (66.8)	285 (63.9)	136 (58.9)	8355 (67.9)	<0.001
Ethnicity, Minorities	1884 (18.9)	348 (21.1)	104 (23.3)	54 (23.4)	2390 (19.4)	0.001
Cardiac diseases						
Cardiomegaly	762 (7.6)	271 (16.5)	123 (27.6)	86 (37.2)	1242 (10.1)	<0.001
Supraventricular arrhythmias	1548 (15.5)	366 (22.2)	102 (22.9)	53 (22.9)	2069 (16.8)	<0.001
CHF	1491 (15.0)	457 (27.7)	192 (43.0)	129 (55.8)	2269 (18.5)	<0.001
Pulmonary heart disease	784 (7.9)	270 (16.4)	117 (26.2)	85 (36.8)	1256 (10.2)	<0.001
AV block	358 (3.6)	61 (3.7)	23 (5.2)	8 (3.5)	450 (3.7)	0.321
Cardiovascular risk factors						
Renal diseases	792 (7.9)	263 (16.0)	95 (21.3)	65 (28.1)	1215 (9.9)	<0.001
Diabetes mellitus	3796 (38.1)	872 (52.9)	270 (60.5)	166 (71.9)	5104 (41.5)	<0.001
Dyslipidemia	8011 (80.3)	1418 (86.1)	395 (88.6)	211 (91.3)	10,035 (81.6)	<0.001
Hypertension	5103 (51.2)	1060 (64.4)	339 (76.0)	200 (86.6)	6702 (54.5)	<0.001
Obesity	2263 (22.7)	353 (21.4)	77 (17.3)	43 (18.6)	2736 (22.2)	0.003
Smoking	4250 (42.6)	736 (44.7)	207 (46.4)	106 (45.9)	5299 (43.1)	0.026
PVD	960 (9.6)	259 (15.7)	89 (20.0)	60 (26.0)	1368 (11.1)	<0.001
Family history of IHD	1008 (10.1)	172 (10.4)	33 (7.4)	13 (5.6)	1226 (10.0)	0.027
Other disorders						
COPD	746 (7.5)	229 (13.9)	87 (19.5)	64 (27.7)	1126 (9.2)	<0.001
Neurological disorders	1559 (15.6)	370 (22.5)	120 (26.9)	77 (33.3)	2126 (17.3)	<0.001
Malignancy	393 (3.9)	67 (4.1)	16 (3.6)	10 (4.3)	486 (4.0)	0.924
Anemia	4080 (40.9)	815 (49.5)	250 (56.1)	134 (58.0)	5279 (42.9)	<0.001
GI bleeding	198 (2.0)	46 (2.8)	13 (2.9)	5 (2.2)	262 (2.1)	0.066
Schizophrenia/Psychosis	182 (1.8)	27 (1.6)	8 (1.8)	7 (3.0)	224 (1.8)	0.574
Alcohol/drug addiction	212 (2.1)	33 (2.0)	8 (1.8)	3 (1.3)	256 (2.1)	0.341
History of malignancy	538 (5.4)	113 (6.9)	36 (8.1)	27 (11.7)	714 (5.8)	<0.001
Clinical characteristics of AMI						
Type of AMI, STEMI	4691 (47.0)	447 (27.1)	78 (17.5)	18 (7.8)	5234 (42.6)	<0.001
Results of echocardiography						
Echocardiography performance	8004 (80.3)	950 (57.7)	236 (52.9)	106 (45.9)	9296 (75.6)	<0.001
Severe LV dysfunction	756 (9.4)	150 (15.8)	64 (27.1)	33 (31.1)	1003 (10.8)	<0.001
LV hypertrophy	451 (5.6)	56 (5.9)	23 (9.7)	11 (10.4)	541 (5.8)	0.004
Mitral regurgitation	389 (4.9)	76 (8.0)	23 (9.7)	11 (10.4)	499 (5.4)	<0.001
Tricuspid regurgitation	282 (3.5)	47 (4.9)	21 (8.9)	10 (9.4)	360 (3.9)	<0.001
Pulmonary hypertension	552 (6.9)	103 (10.8)	41 (17.4)	26 (24.5)	722 (7.8)	<0.001
Results of angiography						
Angiography performance	7096 (71.2)	885 (53.7)	215 (48.2)	93 (40.3)	8289 (67.4)	<0.001
Measure of CAD, No or non-significant	390 (5.5)	37 (4.2)	8 (3.7)	6 (6.5)	441 (5.3)	<0.001
One vessel	2122 (29.9)	185 (20.9)	32 (14.9)	12 (12.9)	2351 (28.4)	
Two vessels	2008 (28.3)	258 (29.2)	58 (27.0)	22 (23.7)	2346 (28.3)	
Three vessels/ LM	2576 (36.3)	405 (45.8)	117 (54.4)	53 (57.0)	3151 (38.0)	
Type of treatment						
Noninvasive	2523 (25.3)	658 (40.0)	208 (46.6)	132 (57.1)	3521 (28.6)	<0.001
PCI	6182 (62.0)	851 (51.7)	213 (47.8)	92 (39.8)	7338 (59.7)	
CABG	1268 (12.7)	138 (8.4)	25 (5.6)	7 (3.0)	1438 (11.7)	

The data are presented as *n* (%) unless otherwise stated. AMI—acute myocardial infarction, AV—Atrioventricular (block), CABG—coronary artery bypass graft, CAD—coronary arteries disease, CHF—congestive heart failure, COPD—chronic obstructive pulmonary disease, GI—gastro-intestinal, IHD—ischemic heart disease, LM—left main (coronary artery), LV—left ventricular, PCI—percutaneous coronary intervention, PVD—peripheral vascular disease, ReAMI—recurrent acute myocardial infarction, SD—standard deviation, STEMI—ST-elevation myocardial infarction.

**Table 2:** Relationship between the number of recurrent acute myocardial infarction (ReAMI) events and the risk of long-term mortality, adjusted for the potential confounders. Multivariable model.

Parameter	B (SE)	HR	(95% CI)	*p*
Number of AMI Events:				
0 (no ReAMI)		1 (ref.)		
2	0.167 (0.039)	1.181	(1.095; 1.274)	<0.001
3	0.309 (0.062)	1.362	(1.207; 1.538)	<0.001
≥3	0.290 (0.079)	1.336	(1.144; 1.561)	<0.001
Age, Years:				
>65		1 (ref.)		
65–75	0.803 (0.044)	2.232	(2.048; 2.434)	<0.001
≥75	1.301 (0.043)	3.672	(3.373; 3.998)	<0.001
Sex, Male vs. Female	−0.067 (0.031)	0.935	(0.881; 0.992)	0.027
Cardiomegaly	0.180 (0.041)	1.197	(1.105; 1.297)	<0.001
Supraventricular arrhythmias	0.203 (0.032)	1.226	(1.150; 1.306)	<0.001
CHF	0.241 (0.033)	1.273	(1.194; 1.357)	<0.001
Pulmonary heart disease	0.094 (0.042)	1.098	(1.011; 1.193)	0.027
Renal diseases	0.388 (0.038)	1.474	(1.367; 1.588)	<0.001
Diabetes mellitus	0.290 (0.03)	1.337	(1.261; 1.417)	<0.001
Dyslipidemia	−0.194 (0.034)	0.823	(0.771; 0.880)	<0.001
Hypertension	−0.076 (0.031)	0.927	(0.873; 0.984)	0.013
Obesity	−0.120 (0.037)	0.887	(0.824; 0.954)	0.001
PVD	0.333 (0.037)	1.395	(1.297; 1.501)	<0.001
Family history of IHD	−0.382 (0.093)	0.682	(0.569; 0.818)	<0.001
COPD	0.525 (0.040)	1.691	(1.565; 1.827)	<0.001
Neurological disorders	0.448 (0.032)	1.564	(1.470; 1.665)	<0.001
Malignancy	0.591 (0.055)	1.806	(1.621; 2.011)	<0.001
Anemia	0.295 (0.031)	1.343	(1.264; 1.427)	<0.001
GI bleeding	0.228 (0.077)	1.256	(1.080; 1.460)	0.003
Schizophrenia/Psychosis	0.435 (0.082)	1.546	(1.316; 1.816)	<0.001
Alcohol/drug addiction	0.550 (0.095)	1.734	(1.439; 2.088)	<0.001
Type of AMI: STEMI vs. NSTEMI	−0.148 (0.034)	0.863	(0.807; 0.922)	<0.001
LOS: >7 days vs. ≤7 days	0.161 (0.031)	1.174	(1.105; 1.247)	<0.001
Type of treatment:				
Noninvasive		1 (ref.)		
PCI	−0.661 (0.035)	0.517	(0.482; 0.553)	<0.001
CABG	−1.055 (0.062)	0.348	(0.308; 0.393)	<0.001
Severe LV dysfunction	0.422 (0.048)	1.526	(1.389; 1.676)	<0.001
LV hypertrophy	0.189 (0.064)	1.208	(1.067; 1.368)	0.003
Mitral regurgitation	0.223 (0.060)	1.250	(1.112; 1.405)	<0.001
Pulmonary hypertension	0.219 (0.055)	1.244	(1.118; 1.385)	<0.001
Year of the index event (one year increase)	−0.013 (0.004)	0.987	(0.980; 0.994)	<0.001

AMI—acute myocardial infarction, B—regression coefficient, CABG—coronary artery bypass graft, CHF—congestive heart failure, CI—confidence interval, COPD—chronic obstructive pulmonary disease, HR—hazard ratio, GI—gastro-intestinal, IHD—ischemic heart disease, LV—left ventricular, NSTEMI—non-ST-elevation myocardial infarction, PCI—percutaneous coronary intervention, PVD—peripheral vascular disease, ReAMI—recurrent acute myocardial infarction, ref.—reference (group), SE—standard deviation, STEMI—ST-elevation myocardial infarction.

## Data Availability

The data presented in this study are available on request from the corresponding author. The data are not publicly available due to privacy.

## Supplementary Materials

The following are available online at https://www.mdpi.com/article/10.3390/jcm10245889/s1. Figure S1: The study flowchart. Table S1: Distribution of the number of recurrent acute myocardial infarction (ReAMI) events in the study population. Table S2: Distribution of the time intervals by the number of recurrent acute myocardial infarction (ReAMI) events. Table S3: The adjusted relative risks (adjusted hazard ratios—AdjHR) for mortality according to the number of recurrent acute myocardial infarction (ReAMI) events and the time intervals. Multivariable model.
