# Supplementary material for: When More Means Less: The Prognosis of Recurrent Acute Myocardial Infarctions
Source: J Clin Med. 2021 Dec 15;10(24):5889. doi: 10.3390/jcm10245889 (PMC8706169; doi:10.3390/jcm10245889)
Supplement: Supplementary file 1 [file jcm-10-05889-s001.zip › jcm-1489851-supplementary.pdf]

Supplementary materials

Figure S1. The study flowchart.

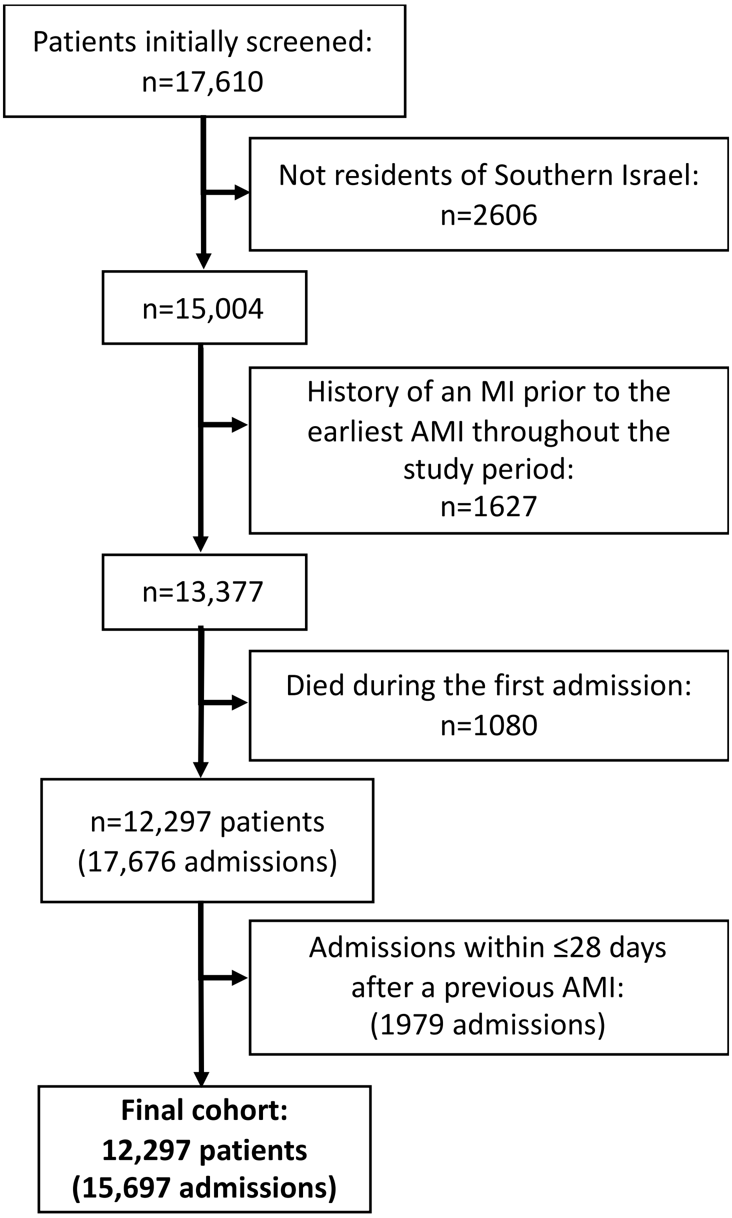

AMI - acute myocardial infarction, MI - myocardial infarction.

Table S1. Distribution of the number of recurrent acute myocardial infarction (ReAMI) events in the study population.

Number of ReAMI events	n (%)
0 (No ReAMI)	9973 (81.1)
1	1647 (13.4)
2	446 (3.6)
3	136 (1.1)
4	51 (0.4)
5	26 (0.2)
6	9 (0.1)
7	7 (0.1)
8	2 (0)

ReAMI - recurrent acute myocardial infarction.

Table S2. Distribution of the time intervals by the number of recurrent acute myocardial infarction (ReAMI) events.

**Time interval (years)**	**<0.5**	**1**	**2**	**3**	**4**	**≥5**	**Total**
**Number of ReAMI events**							
0 (No ReAMI)							9973 (100)
1	386 (23.4)	170 (10.3)	275 (16.7)	152 (9.2)	123 (7.5)	541 (32.8)	1647 (100)
≥2	24 (3.5)	56 (8.3)	74 (10.9)	89 (13.1)	74 (10.9)	360 (53.2)	677 (100)

The data are presented as n (%).

ReAMI - recurrent acute myocardial infarction.

Table S3. The adjusted relative risks (adjusted hazard ratios – adjHR) for mortality according to the number of recurrent acute myocardial infarction (ReAMI) events and the time intervals. Multivariable model.

Parameter	B (SE)	HR	(95% CI)	p
**Number of ReAMI events and time interval (years)**				
**0 (No ReAMI)**		**1 (ref.)**		
**1:**				
**<0.5**	**0.104 (0.069)**	**1.110**	**(0.969 ; 1.271)**	**0.131**
**1**	**0.170 (0.096)**	**1.185**	**(0.981 ; 1.432)**	**0.078**
**2**	**0.260 (0.080)**	**1.297**	**(1.110 ; 1.517)**	**0.001**
**3**	**0.101 (0.104)**	**1.106**	**(0.902 ; 1.358)**	**0.333**
**4**	**0.119 (0.122)**	**1.127**	**(0.888 ; 1.431)**	**0.327**
**≥5**	**0.204 (0.068)**	**1.226**	**(1.074 ; 1.400)**	**0.003**
**≥2:**				
**<0.5**	**0.791 (0.225)**	**2.205**	**(1.418 ; 3.429)**	**<0.001**
**1**	**0.659 (0.147)**	**1.933**	**(1.448 ; 2.580)**	**<0.001**
**2**	**0.147 (0.135)**	**1.158**	**(0.889 ; 1.509)**	**0.276**
**3**	**0.137 (0.119)**	**1.147**	**(0.908 ; 1.448)**	**0.250**
**4**	**0.408 (0.136)**	**1.503**	**(1.152 ; 1.961)**	**0.003**
**≥5**	**0.280 (0.071)**	**1.323**	**(1.150 ; 1.521)**	**<0.001**
Age, Years:				
>65		1 (ref.)		
65-75	0.801 (0.044)	2.227	(2.042 ; 2.428)	<0.001
≥75	1.299 (0.043)	3.665	(3.366 ; 3.991)	<0.001
Sex, Male vs. Female	-0.069 (0.031)	0.933	(0.879 ; 0.991)	0.024
Cardiomegaly	0.185 (0.041)	1.203	(1.110 ; 1.304)	<0.001

Supraventricular arrhythmias	0.206 (0.032)	1.229	(1.153 ; 1.309)	<0.001
CHF	0.242 (0.033)	1.274	(1.195 ; 1.359)	<0.001
Pulmonary heart disease	0.093 (0.042)	1.097	(1.009 ; 1.192)	0.029
Renal diseases	0.392 (0.038)	1.480	(1.373 ; 1.596)	<0.001
Diabetes mellitus	0.286 (0.030)	1.332	(1.256 ; 1.412)	<0.001
Dyslipidemia	-0.198 (0.034)	0.820	(0.768 ; 0.877)	<0.001
Hypertension	-0.073 (0.031)	0.929	(0.875 ; 0.987)	0.017
Obesity	-0.124 (0.037)	0.884	(0.821 ; 0.951)	0.001
PVD	0.334 (0.037)	1.396	(1.298 ; 1.503)	<0.001
Family history of IHD	-0.382 (0.093)	0.683	(0.569 ; 0.819)	<0.001
COPD	0.522 (0.040)	1.685	(1.559 ; 1.821)	<0.001
Neurological disorders	0.449 (0.032)	1.567	(1.473 ; 1.668)	<0.001
Malignancy	0.600 (0.055)	1.822	(1.635 ; 2.030)	<0.001
Anemia	0.294 (0.031)	1.341	(1.262 ; 1.426)	<0.001
GI bleeding	0.229 (0.077)	1.257	(1.081 ; 1.461)	0.003
Schizophrenia/Psychosis	0.436 (0.082)	1.546	(1.316 ; 1.816)	<0.001
Alcohol/drug addiction	0.550 (0.095)	1.734	(1.439 ; 2.089)	<0.001
Type of AMI: STEMI vs. NSTEMI	-0.150 (0.034)	0.861	(0.805 ; 0.920)	<0.001
LOS: >7 days vs. ≤7 days	0.158 (0.031)	1.171	(1.102 ; 1.244)	<0.001
Type of treatment:				
Noninvasive		1 (ref.)		
PCI	-0.663 (0.035)	0.515	(0.481 ; 0.552)	<0.001
CABG	-1.052 (0.062)	0.349	(0.309 ; 0.394)	<0.001
Severe LV dysfunction	0.424 (0.048)	1.529	(1.391 ; 1.679)	<0.001
LV hypertrophy	0.190 (0.064)	1.209	(1.068 ; 1.370)	0.003
Mitral regurgitation	0.226 (0.060)	1.253	(1.115 ; 1.409)	<0.001
Pulmonary hypertension	0.220 (0.055)	1.246	(1.120 ; 1.387)	<0.001
Year of the index event (one year increase)	-0.013 (0.004)	0.987	(0.980 ; 0.994)	<0.001

AMI - acute myocardial infarction, B – regression coefficient, CABG - coronary artery bypass graft, CHF - congestive heart failure, CI – confidence interval, COPD – chronic obstructive pulmonary disease, HR – hazard ratio, GI – gastro-intestinal, IHD - ischemic heart disease, LOS – length of (hospital) stay, LV - left ventricular, NSTEMI – non-ST-elevation myocardial infarction, PCI - percutaneous coronary intervention, PVD - peripheral vascular disease, ReAMI - recurrent acute myocardial infarction, ref. – reference (group), SE – standard error, STEMI – ST-elevation myocardial infarction.
